# Vascular risk factors and staging of atherosclerosis in patients and controls: The Norwegian Stroke in the Young Study

**DOI:** 10.1177/23969873221098582

**Published:** 2022-05-10

**Authors:** Beenish Nawaz, Annette Fromm, Halvor Øygarden, Geir Egil Eide, Sahrai Saeed, Rudy Meijer, Michiel L Bots, Kristin Modalsli Sand, Lars Thomassen, Halvor Næss, Ulrike Waje-Andreassen

**Affiliations:** 1 Department of Clinical Medicine 1, University of Bergen, Bergen, Norway; 2 Department of Neurology, Haukeland University Hospital, Bergen, Norway; 3 Department of Neurology, Sørlandet Hospital, Kristiansand, Norway; 4 Department of Health and Nursing Sciences, University of Agder, Kristiansand, Norway; 5 Centre for Clinical Research, Haukeland University Hospital, Bergen, Norway; 6 Department of Global Public Health and Primary Care, University of Bergen, Bergen, Norway; 7 Department of Cardiology, Haukeland University Hospital, Bergen, Norway; 8 Julius Center of Health Sciences and Primary Care, University Medical Center Utrecht, Utrecht University, Utrecht, The Netherlands; 9 Department of Medicine, Sørlandet Hospital, Flekkefjord, Norway; 10 The Institute of Health and Society, Faculty of Medicine, University of Oslo, Oslo, Norway

**Keywords:** Young ischaemic stroke, vascular risk factors, fat measurements, EAT, VAT, SAT, staging of atherosclerosis

## Abstract

**Objectives:**

We studied the prevalence of vascular risk factors (RFs) among 385 ischaemic stroke patients ⩽60 years and 260 controls, and their association with atherosclerosis in seven vascular areas.

**Methods:**

History of cardiovascular events (CVE), hypertension, diabetes mellitus (DM), dyslipidaemia, pack-years of smoking (PYS), alcohol, and physical inactivity were noted. Blood pressure, body mass index (BMI), waist-hip ratio (WHR), lipid profile, epicardial adipose tissue (EAT), visceral abdominal adipose tissue (VAT), and subcutaneous abdominal adipose tissue were measured. Numeric staging of atherosclerosis was done by standardized examination of seven vascular areas by right and left carotid and femoral intima-media thickness, electrocardiogram, abdominal aorta plaques, and the ankle-arm index. All results were age and sex-adjusted. Poisson regression analysis was applied.

**Results:**

At age ⩽49 years at least one RF was present in 95.6% patients versus 90.0% controls. Compared to controls, male patients and middle-aged female patients showed no significant differences. Young female patients compared to young female controls had a higher burden of RFs (94.3% vs 88.6%, *p* = 0.049). Poisson regression analysis combined for patients and controls, adjusted for age and sex, showed numeric staging of atherosclerosis associated with age, prior CVE, hypertension, DM, dyslipidaemia, PYS, alcohol, BMI, WHR, EAT, VAT, and an increased number of risk factors. Adjusted for all risk factors, numeric staging of atherosclerosis was associated with increasing age, hypertension, DM, PYS, and BMI.

**Conclusion:**

Vascular risk factors are highly prevalent in young- and middle-aged patients and controls, and are predictors of established atherosclerosis at study inclusion. Focus on main modifiable vascular RFs in primary prevention, and early and aggressive secondary treatment of patients are necessary to reduce further progression of atherosclerosis.

## Introduction

According to TOAST,^1^ the internationally widely applied classification of causes of stroke, we found large artery atherosclerosis due to 50% arterial stenosis prevalent among our patients in 7.3%, and cryptogenic stroke prevalent in 37.1%.^2^ We found total prevalent atherosclerosis among 61.4% of our stroke patients, and even our patients ⩽49 years with cryptogenic stroke had total prevalent atherosclerosis among 71.4% males and 37.5% females.^2^ Young stroke patients are at substantial risk for new cardiovascular events (CVE) and death, particularly due to atherosclerosis leading to coronary artery disease (CAD), mostly attributable to modifiable risk factors (RFs).^3,4^ The number of RFs is associated with increased risk for CVE and mortality,^5^ and the RF burden is high throughout Europe.^6^

Carotid and coronary arteries, the abdominal aorta, and femoral arteries are particularly susceptible areas for atherosclerosis. Many previous studies have related localized atherosclerosis at one single vascular area to vascular RFs,^7–10^ and to CVE and mortality.^9,11,12^ However, young stroke studies on atherosclerosis at multiple vascular areas are scarce. A previous study of 212 patients without known cardiovascular disease (CVD) showed high presence of subclinical atherosclerosis in carotid, femoral and coronary areas, but a weak concordance between the different vascular territories, and suggested that all three vascular areas should be investigated.^13^ Post-mortem studies of subjects indicated that atherosclerosis developed simultaneously in cerebrovascular, coronary and peripheral arteries, and a weak positive correlation was found between femoral and coronary atherosclerosis,^14,15^ whereas clinical studies of patients showed a strong positive correlation between femoral atherosclerosis and the severity of CAD.^16,17^ Numerous studies also reported a modest relation between carotid intima-media thickness and CAD, which probably reflects variability in the atherosclerotic process between different vascular areas.^18^

Analog to oncological staging of tumors, we performed numerical staging of atherosclerosis, and related the findings to vascular risk factors among patients with ischaemic stroke and their partners as controls as part of a three-generation research program. Due to the fact that CVD dominates causes of hospital admission and mortality in Norway,^19,20^ as in other industrialized countries,^21,22^ inclusion of controls was wanted as previous studies have shown that atherosclerosis starts early in life, and has a high prevalence at a subclinical level.^23–25^ Based on our previous findings of prevalence of atherosclerosis, showing only a significant difference for young female patients compared to young female controls,^2^ our hypothesis was that a high number of risk factors are prevalent in both groups, but even more among patients, and we aimed to identify the predictors of severity of atherosclerosis.

## Methods

### Study population

NOR-SYS 3-generation design and ultrasound protocol, methods and results of inclusion have been published.^26,27^ In total, 152 young (⩽49 years) and 233 middle-aged (50–60 years) acute ischaemic stroke patients and 260 partners/ex-partners, serving as controls, were included from 2010 to 2015. Supplemental Figure 1 shows a chart with inclusion and exclusion criteria and methods used for patients and controls.

### Consents and ethics

The study complies with the Declaration of Helsinki, is approved by the Regional Ethics Committee (REK-Vest 2010/74), and registered in ClinicalTrials.gov (NCT01597453). Written consent is present for all study participants.

### Risk factor definitions

Vascular RFs included prior CVE, hypertension, diabetes mellitus (DM), dyslipidaemia, pack-years of smoking (PYS), alcohol, physical inactivity, body mass index (BMI), waist-hip ratio (WHR), epicardial adipose tissue (EAT), visceral abdominal adipose tissue (VAT), and subcutaneous abdominal adipose tissue (SAT).

Prior CVEs including stroke, CAD, and peripheral artery disease (PAD) were verified in hospital records. CAD included cases of myocardial infarction and/or verified CAD by percutaneous coronary intervention (PCI). Angina pectoris or non-obstructive CAD were not included. Uncertain information about previous stroke was substantiated by information from cerebral magnetic resonance imaging. Hypertension, DM, and dyslipidaemia were defined as known when treated by lifestyle changes and/or by medication before admission, or when diagnosed during hospitalization. Hypertension was diagnosed if blood pressure was >140/90 mmHg in two separate measurements in both arms after 15–30 min rest in a supine position following the ultrasound examination. In patients only, DM and dyslipidaemia were diagnosed by blood tests as HbA1c >6.4%, total cholesterol >5.0 mmol/L, low-density lipoprotein >3.0 mmol/L, high density lipoprotein <1.0 mmol/L, and/or triglycerides >2.5 mmol/L. In controls, an unknown history of dyslipidaemia was categorized as normal. Smoking was categorized as never-smoking, ex-smoking when stopped at least 1 year ago, and current smoking, and pack-years of smoking (PYS) were calculated as number of cigarette packs (20 cig/pack) per day multiplied by number of years smoking. Alcohol consumption was categorized as increased if ⩾12 units/week. Physical inactivity was defined as activity of light, moderate or vigorous intensity of less than 60 min/week. BMI was categorized as increased if ⩾25 kg/m^2^. Increased WHR was defined for females (⩾0.85) and males (⩾0.90).^28^ Our methods for ultrasonographic measurements of EAT, VAT, and SAT have been published.^26^ EAT thickness with a cut-off value of 0.5 cm was used to identify individuals at higher cardiovascular risk.^29^ Cut-off values for ultrasonographic abdominal fat measurements are not yet established. Thus, we based our sex-specific high VAT and SAT definitions on 90th percentile cut-points from normal weight referent sample, as a similar method was used in the Framingham heart study.^30^ The referent sample in our study consisted of 133 men and 99 women with normal BMI, and our cut-offs of VAT and SAT were 9.6 and 3.5 cm in men and 8.3 and 4.2 cm in women, respectively. The total risk factor burden (RFB) was assessed as number of RFs present (0–12).

### Staging of atherosclerosis

Staging of atherosclerosis among patients and controls, was defined by the number of affected vascular areas from 0 to 7, based on right and left mean carotid and femoral intima-media thickness ⩾1.5 mm respectively, ischemic electrocardiogram (ECG), presence of abdominal aorta plaques and ankle-arm index ⩽0.9. For carotid intima-media measurements (IMT), we used the maximum value of a total of 12 standardized artery segments at the far wall; 4 of the distal common carotid artery, 1 of the carotid bifurcation, and 1 of the proximal internal carotid artery, measured on the right and the left side. Plaques were included, and defined as focal IMT⩾1.5 mm. For femoral IMT measurements, we used the maximum value of a total of four standardized artery segments at the far wall of the common femoral artery and the superficial femoral artery on the right and the left side. Any artery segment was measured over a distance of 1 cm, resulting in a mean value of about 100 possible point-to point measurements, calculated by Philips Q-Lab software^®^ (Advanced Ultrasound Quantification, Philips Ultrasound, Bothell, WA, USA). Detailed procedures of our ultrasound protocol have been published.^26^ We chose not to include intracranial arterial pathology analysis due to uncertainties in defining the cause and degree of stenosis by common imaging methods.^31^

### Statistics

The mean and standard deviation (SD) were used for descriptive statistics, all adjusted by age and sex. Univariate comparisons of RFs between patients and controls were done within the four age and sex strata using unpaired *t*-test and the Fisher’s exact test. For unadjusted comparison of all patients to their individual partners/ex-partners, McNemar’s test of symmetry was used. In the Poisson regression analysis, we related possible risk factors to the results of numeric staging of atherosclerosis for patients and controls separately as our controls consisted of patients’ partners and ex-partners (Table 2). However, as predictors of severity of atherosclerosis, we combined both groups in Table 3. To adjust for confounding, Poisson regression analysis was done with respect to age, sex, and all RFs. Interactions were tested for association of risk factors on atherosclerosis between patients and controls. Results were reported as incidence rate ratios (IRR) with 95% confidence intervals (CI). Two-sided *p*-values ⩽0.05 were considered significant. All statistical analyses were performed in Stata SE 17.0.

## Results

### Study population

At inclusion, patients had a mean age of 49.5 (range 15–60) years and controls had a mean age of 50.3 (range 21–69) years (Table 1). The majority of patients were males (68.6%), and the majority of controls were females (70.0%). Young age ⩽49 years was present for 39.5% of patients and 39.2% of controls.

**Table 1.** Baseline characteristics of 385 acute ischemic stroke patients and 260 controls.

Variables		NA, *n*	All	Male ⩽49 years	Male ⩾50 years	Female ⩽49 years	Female ⩾50 years
Categories							
Patients, *n* (%)	P	0	385 (100.0)	94 (24.4)	170 (44.2)	58 (15.1)	63 (16.4)
Controls, *n* (%)	C	0	260 (100.0)	28 (10.8)	50 (19.2)	74 (28.5)	108 (41.5)
Age (years), mean (SD)	P	0	49.5 (9.8)	40.5 (8.2)	55.9 (3.0)	38.4 (8.9)	55.8 (2.9)
	C	0	50.3 (8.6)	42.0 (6.7)	57.5 (4.9)	41.7 (6.6)	55.0 (3.2)
Unpaired *T*-test, *p*^a^			0.001*	0.312	0.028	0.019	0.102
Prior CVE, *n* (%)	P	0	49 (12.7)	8 (8.5)	27 (15.9)	5 (8.6)	9 (14.3)
	C	0	18 (6.9)	3 (10.7)	8 (16.0)	0 (0.0)	7 (6.5)
Fisher’s exact test, *p*			0.121**	0.715	1.000	0.015	0.107
Prior stroke^b^, *n* (%)	P	0	27 (7.0)	7 (7.4)	10 (5.9)	2 (3.4)	8 (12.7)
	C	0	8 (3.1)	0 (0.0)	6 (12.0)	0 (0.0)	2 (1.9)
Fisher’s exact test, *p*			0.263**	0.199	0.210	0.191	0.006
Prior CHD, *n (%)*	P	0	25 (6.5)	3 (3.2)	16 (9.4)	3 (5.2)	3 (4.8)
	C	0	8 (3.1)	3 (10.7)	2 (4.0)	0 (0.0)	3 (2.8)
Fisher’s exact, *p*			0.119**	0.136	0.377	0.082	0.671
Prior PAD, *n* (%)	P	0	4 (1.0)	0 (0.0)	4 (2.4)	0 (0.0)	0 (0.0)
	C	0	4 (1.5)	0 (0.0)	1 (2.0)	0 (0.0)	3 (2.8)
Fisher’s exact, *p*			1.000**	–	1.000	–	0.298
Hypertension^c^, *n* (%)	P	0	238 (61.8)	46 (48.9)	125 (73.5)	19 (32.8)	48 (76.2)
	C	1	89 (34.4)	5 (17.9)	31 (62.0)	13 (17.6)	40 (37.4)
Fisher’s exact, *p*			<0.001**	0.004	0.156	0.065	<0.001
Diabetes mellitus^d^, *n* (%)	P	0	44 (11.4)	6 (6.4)	28 (16.5)	5 (8.6)	5 (7.9)
	C	0	15 (5.8)	1 (3.6)	6 (12.0)	2 (2.7)	6 (5.6)
Fisher’s exact, *p*			0.071**	1.000	0.512	0.239	0.536
Dyslipidaemia^e^, *n* (%)	P	0	293 (76.1)	61 (64.9)	145 (85.3)	35 (60.3)	52 (82.5)
	C	0	34 (13.1)	3 (10.7)	11 (22.0)	3 (4.1)	17 (15.7)
Fisher’s exact, *p*			<0.001**	<0.001	<0.001	<0.001	<0.001
Smoking^f^, *n* (%)	P	0	268 (69.6)	59 (62.8)	130 (76.5)	33 (56.9)	46 (73.0)
	C	1	169 (65.3)	17 (60.7)	37 (74.0)	41 (56.2)	74 (68.5)
Fisher’s exact, *p*			0.313**	1.000	0.710	1.000	0.605
Alcohol^g, *n* (%)^	P	4	38 (10.0)	12 (12.9)	22 (13.1)	1 (1.8)	3 (4.8)
	C	2	8 (3.1)	2 (7.1)	3 (6.0)	1 (1.4)	2 (1.9)
Fisher’s exact, *p*			0.007**	0.516	0.211	1.000	0.359
Physical inactivity^h^, *n* (%)	P	0	71 (18.4)	21 (22.3)	33 (19.4)	7 (12.1)	10 (15.9)
	C	0	28 (10.8)	4 (14.3)	8 (16.0)	10 (13.5)	6 (5.6)
Fisher’s exact, *p*			0.134	0.432	0.683	1.000	0.031
BMI ⩾25 kg/m^2^, *n* (%)	P	3	254 (66.5)	66 (70.2)	123 (72.8)	31 (55.4)	34 (54.0)
	C	4	152 (59.4)	20 (74.1)	32 (64.0)	45 (61.6)	55 (51.9)
Fisher’s exact, *p*			0.028**	0.811	0.288	0.477	0.874
Increased WHR^i, *n* (%)^	P	26	254 (70.8)	56 (64.4)	131 (82.9)	27 (50.0)	40 (66.7)
	C	5	130 (51.0)	13 (48.1)	40 (81.6)	28 (38.9)	49 (45.8)
Fisher’s exact, *p*			<0.001**	0.176	0.831	0.276	0.010
EAT mean in cm (SD)	P	13	0.63 (0.23)	0.58 (0.20)	0.67 (0.24)	0.54 (0.16)	0.68 (0.28)
	C	1	0.59 (0.20)	0.54 (0.24)	0.59 (0.17)	0.54 (0.22)	0.65 (0.19)
Unpaired T-test, p			0.004*	0.367	0.022	0.956	0.329
VAT mean in cm (SD)	P	9	9.40 (2.49)	9.44 (2.17)	10.3 (2.39)	7.6 (2.20)	8.66 (2.38)
	C	1	8.02 (2.43)	8.64 (2.59)	9.78 (2.42)	7.18 (2.35)	7.62 (2.00)
Unpaired *T*-test, *p*			<0.001*	0.118	0.217	0.297	0.003
SAT mean in cm (SD)	P	9	3.25 (1.33)	3.15 (1.35)	2.95 (1.10)	3.71 (1.63)	3.81 (1.27)
	C	1	3.40 (1.24)	3.25 (1.18)	2.92 (1.15)	3.59 (1.35)	3.54 (1.17)
Unpaired *T*-test, *p*			0.120*	0.704	0.888	0.650	0.165

NA: not available; *n*: number of observations; P: patients; C: controls; SD: standard deviation; CVE: cardiovascular events; CHD: coronary heart disease; PAD: peripheral artery disease; BMI: body mass index; WHR: waist-hip ratio; EAT: epicardial adipose tissue; cm: centimetre; VAT/SAT: visceral/subcutaneous abdominal adipose tissue.

a *p*-Values are comparisons between patients and controls, and significant p-values are given in bold.

b Two patients and one control had haemorrhagic stroke.

c Hypertension was defined as known, or diagnosed if blood pressure >140/90 mmHg.

d Diabetes mellitus was defined as known among patients and controls, or diagnosed by HbA1c >6.5% among patients only.

e Dyslipidaemia was defined as known among patients and controls, or diagnosed by blood tests among patients only.

f Smoking included ex-smokers and active smokers.

g Alcohol intake is defined as ⩾12 units/week.

h Physical inactivity is defined as activity less than 60 min/week.

i Increased WHR is defined as ⩾0.85 in females and ⩾0.90 in male.

### Reported risk factors, clinical, and ultrasonographic findings among young study participants ⩽49 years

Compared to controls, young male patients had higher prevalence of hypertension (48.9% vs 17.9%; *p* = 0.004), and young female patients had higher prevalence of prior CVE (8.6% vs 0.0%; *p* = 0.015) (Table 1).

Obesity (BMI ⩾25.0 kg/m^2^ 64.7%, increased WHR 58.9%, increased EAT 51.3%), dyslipidaemia (63.2%), smoking (60.5%), and hypertension (42.8%) were the most frequent vascular RFs among young stroke patients (Supplemental Table 1). Regarding sex differences, young male patients had more prevalent hypertension (48.9% vs 32.8%, *p* = 0.050), and higher alcohol intake (12.9% vs 1.8%, *p* = 0.018) than young female patients. Prevalence of all RFs was increased among middle-aged patients, except from alcohol intake, physical inactivity, BMI, and SAT (Supplemental Table 1).

The most common RFs among couples were smoking and physical inactivity (Supplemental Table 2).

### Missing data

WHR was not obtained for 26 stroke patients, mainly due to their disability. Among them, 18 patients had increased BMI. Ultrasonographic measurements were not obtained for two patients due to unconsciousness at admission, and morbid obesity causing insufficient imaging quality, respectively. Some EAT, VAT, and SAT had missing segments due to bad imaging quality. Due to missing information of some risk factors and vascular areas, the RFB was assessed in 346 patients and 250 controls, and atherosclerotic staging was performed in 324 patients and 238 controls. The Poisson regression analysis was possible to perform for 307 (94.8%) of 324 patients, and 232 (97.5%) of 238 controls.

### Total risk factor burden

**Figure 1.**
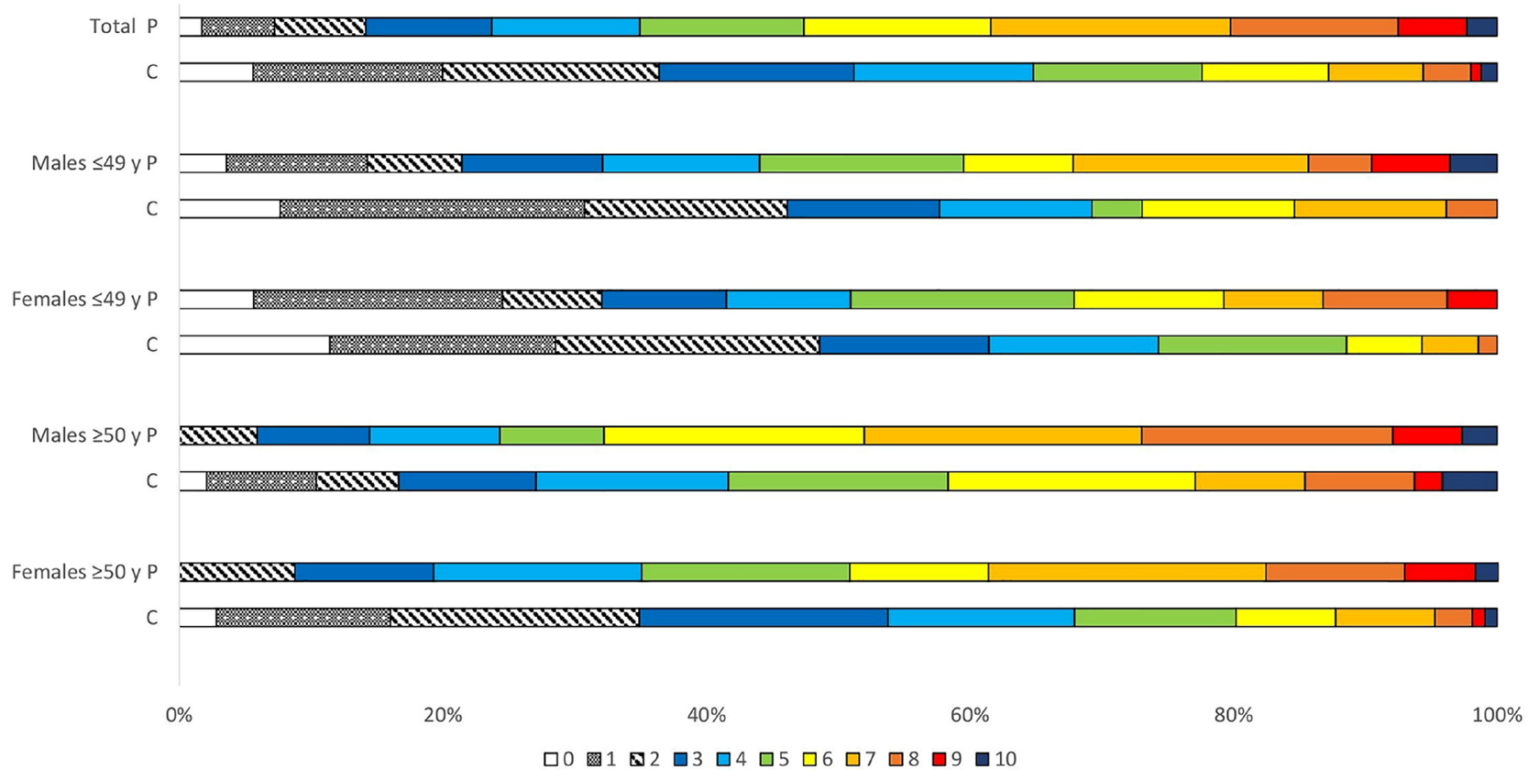
Total burden of risk factors^a^ among 346 patients and 250 controls. ^a^Risk factors included prior cardiovascular events, hypertension, diabetes mellitus, dyslipidaemia, smoking, alcohol intake, physical inactivity, body mass index, waist-hip ratio, epicardial adipose tissue, visceral abdominal adipose tissue, and subcutaneous abdominal adipose tissue. P: patients; C: controls; y: years.

The RFB was higher in young female patients compared to young female controls (94.3% vs 88.6%, *p* = 0.049). Among subgroup analysis of middle-aged females, young males, and middle-aged males, there were no significant differences between patients and controls (100.0% vs 97.2%, *p* = 0.689; 96.4% vs 92.3%, *p* = 0.590; and 100.0% vs 97.9%, *p* = 0.240, respectively), as presented in Figure 1. The RFB was higher in middle-aged male patients than in young male patients (100.0% vs 96.4%, *p* < 0.001) and in middle-aged female patients than in young female patients (100.0% vs 94.3%, *p* = 0.016). There were no sex differences in the RFB among stroke patients (males 98.7% vs females 97.3% *p* = 0.267).

### Risk of atherosclerosis

Poisson regression analysis, separate for patients and controls, adjusted for age and sex in Table 2 showed that age, sex, prior CVE, hypertension, DM, dyslipidaemia, PYS, alcohol, physical inactivity, BMI, EAT, VAT, and an increased number of RFs were associated with numeric staging of atherosclerosis among patients, and age, sex, prior CVE, PYS, physical inactivity, BMI, WHR, EAT, VAT, and an increased number of RFs were associated with numeric staging of atherosclerosis among controls. Adjusted for all risk factors, numeric staging of atherosclerosis was associated with age, DM, and PYS among patients and age, PYS, and alcohol among controls.

**Table 2.** Poisson regression of possible associated risk factors with the number of areas^a^ with atherosclerosis among 324 patients and 238 controls.

Group	*n*	Adjusted for age and sex			Adjusted for all variables		
Variables		IRR	95% CI	*p*-Value	IRR	95% CI	*p*-Value
Patients						*n* = 307	
Age per 10 years	324	1.93	(1.68, 2.22)	<0.001	1.60	(1.36, 1.89)	<0.001
Sex (male)	324	1.36	(1.10, 1.69)	0.005	1.24	(0.95, 1.61)	0.118
Prior CVE^b^	324	1.63	(1.30, 2.06)	<0.001	1.23	(0.91, 1.67)	0.181
Hypertension	324	1.67	(1.34, 2.09)	<0.001	1.30	(0.98, 1.73)	0.071^ǂ^
Diabetes mellitus	324	1.50	(1.15, 1.94)	0.002	1.44	(1.03, 2.02)	0.034
Dyslipidaemia	324	1.40	(1.08, 1.81)	0.012	1.19	(0.87, 1.61)	0.274
Pack-years of smoking^c^	324	1.02	(1.02, 1.03)	<0.001	1.02	(1.02, 1.03)	<0.001
Increased alcohol intake^d^	321	1.49	(1.15, 1.92)	0.002	1.17	(0.85, 1.60)	0.329
Physical inactivity^e^	324	1.46	(1.18, 1.81)	<0.001	1.17	(0.87, 1.59)	0.299
BMI ⩾25 kg/m^2^	324	1.39	(1.13, 1.72)	0.002	1.14	(0.82, 1.60)	0.438
Increased WHR^f)^	312	1.24	(0.98, 1.57)	0.070	0.74	(0.52, 1.06)	0.104
EAT (cm)	322	2.22	(1.55, 3.16)	<0.001	1.41	(0.94, 2.11)	0.101^ǂ^
VAT (cm)	324	1.06	(1.02, 1.11)	0.004	1.01	(0.95, 1.07)	0.697
SAT (cm)	324	1.03	(0.95, 1.18)	0.463	1.04	(0.94, 1.15)	0.451
Number of risk factors	307	1.17	(1.12, 1.22)	<0.001	1.04	(0.89, 1.21)	0.624
Controls						*n* = 232	
Age at inclusion (years)	238	2.22	(1.79, 2.76)	<0.001	1.36	(1.03, 2.29)	<0.001
Sex (male)	238	1.59	(1.17, 2.15)	0.003	1.43	(0.99, 2.04)	0.054
Prior CVE^b^	238	1.86	(1.26, 2.74)	0.002	1.49	(0.83, 2.64)	0.177
Hypertension	238	1.33	(0.98, 1.81)	0.067	1.17	(0.75, 1.83)	0.491
Diabetes mellitus	238	1.21	(0.65, 1.94)	0.680	1.02	(0.50, 2.09)	0.953
Dyslipidaemia	238	1.13	(0.78, 1.64)	0.510	0.88	(0.52, 1.50)	0.641
Pack-years of smoking^c^	238	1.03	(1.02, 1.04)	<0.001	1.04	(1.02, 1.05)	<0.001
Increased alcohol intake^d^	237	1.00	(0.52, 1.92)	0.997	0.20	(0.07, 0.53)	0.001
Physical inactivity^e^	238	1.61	(1.10, 2.36)	0.013	1.00	(0.58, 1.73)	0.997
BMI ⩾25 kg/m^2^	237	1.52	(1.12, 2.07)	0.008	0.92	(0.54, 1.56)	0.751
Increased WHR^f)^	235	1.60	(1.16, 2.21)	0.004	1.21	(0.75, 1.94)	0.430
EAT (cm)	237	2.42	(1.20, 4.90)	0.014	1.44	(0.54, 3.87)	0.465
VAT (cm)	238	1.07	(1.01, 1.14)	0.024	0.94	(0.84, 1.04)	0.218
SAT (cm)	238	1.12	(0.98, 1.27)	0.087	1.06	(0.88, 1.28)	0.517
Number of risk factors	232	1.14	(1.07, 1.21)	<0.001	1.10	(0.80, 1.41)	0.675

*n*: number of observations; IRR: incidence rate ratio; CI: confidence interval; CVE: cardiovascular events; BMI: body mass index; WHR: waist-hip ratio; EAT: epicardial adipose tissue; cm: centimetre; VAT/SAT: visceral/subcutaneous abdominal adipose tissue.

Significant *p*-values are given in bold. The final regression analysis with adjustment of all variables was also performed after removal of the variable “number of risk factors” among 307 patients and 232 controls. This was performed as the actual final regression analysis included the risk factors twice – firstly as individual risk factors and secondly in the variable “number of risk factors.”

ǂ Risk factor which then became significant.

a Seven vascular areas were assessed for atherosclerosis by electrocardiogram, ankle-arm index, right and left carotid and femoral intima-media thickness and abdominal aorta plaques.

b CVE included stroke, coronary artery disease or peripheral artery disease.

c Pack-years of smoking was calculated as number of cigarette packs (20 cig/pack) per day multiplied by number of years smoking.

d Increased alcohol intake is defined as >12 units/week.

e Physical inactivity was defined as activity less than 60 min/week.

f Increased WHR is defined as ⩾0.85 in females and ⩾0.90 in males.

The Poisson regression analysis combined for patients and controls, adjusted for age, sex, and group (patients vs controls) in Table 3, showed that age, prior CVE, hypertension, DM, dyslipidaemia, PYS, alcohol, BMI, WHR, EAT, VAT, and an increased number of RFs were associated with numeric staging of atherosclerosis. After adjustment for all risk factors, age, hypertension, DM, PYS, and BMI were associated with numeric staging of atherosclerosis.

**Table 3.** Poisson regression of possible associated risk factors with the number of areas^a^ with atherosclerosis among 562 study participants (324 patients and 238 controls).

Group	*n*	Adjusted for age, sex, and group			Adjusted for all variables		
Variables		IRR	95% CI	*p*-Value	IRR	95% CI	*p*-Value
Patients and controls						*n* = 423	
Group (patient vs control)	503	0.88	(0.74, 1.05)	0.151	1.09	(0.85, 1.38)	0.502
Age per 10 years	503	1.82	(1.63, 2.03)	<0.001*	1.50	(1.31, 1.72)	<0.001*
Sex (male)	503	0.92	(0.78, 1.09)	0.329*	0.81	(0.66, 1.01)	0.057^ǂ^*
Prior CVE^b^	503	1.54	(1.25, 1.91)	<0.001*	1.14	(0.86, 1.51)	0.352*
Hypertension	502	1.41	(1.19, 1.67)	<0.001*	1.34	(1.04, 1.71)	0.022*
Diabetes mellitus	503	1.53	(1.22, 1.93)	<0.001	1.51	(1.12, 2.03)	0.007
Dyslipidaemia	503	1.36	(1.10, 1.67)	0.004	1.07	(0.83, 1.38)	0.595
Pack-years of smoking^c^	502	1.02	(1.02, 1.02)	<0.001*	1.02	(1.02, 1.03)	<0.001*
Increased alcohol intake^d^	498	1.35	(1.74, 1.92)	0.022 *	1.11	(0.82, 1.50)	0.515*
Physical inactivity^e^	503	1.22	(1.00, 1.49)	0.053*	0.99	(0.75, 1.31)	0.947*
BMI ⩾25 kg/m^2^	501	1.57	(1.32, 1.86)	<0.001	1.49	(1.12, 1.98)	0.006
Increased WHR^f^	491	1.41	(1.18, 1.69)	<0.001	0.97	(0.73, 1.30)	0.854
EAT (cm)	500	1.70	(1.23, 2.35)	0.001*	1.17	(0.78, 1.75)	0.453*
VAT (cm)	503	1.06	(1.03, 1.10)	0.001*	0.97	(0.92, 1.03)	0.345*
SAT (cm)	503	1.05	(0.99, 1.12)	0.115	1.02	(0.94, 1.12)	0.591*
Number of risk factors	423	1.16	(1.12, 1.21)	<0.001 *	1.03	(0.90, 1.38)	0.700*

*n*: number of observations; IRR: incidence rate ratio; CI: confidence interval; CVE: cardiovascular events; BMI: body mass index; WHR: waist hip ratio; EAT: epicardial adipose tissue; cm: centimetre; VAT/SAT: visceral/subcutaneous abdominal adipose tissue.

Significant *p*-values are given in bold. The final regression analysis with adjustment of all variables was also performed after removal of the variable “number of risk factors” 481 study participants. This was performed as the actual final regression analysis included the risk factors twice – firstly as individual risk factors and secondly in the variable “number of risk factors.”

ǂ Risk factor which then became significant.

* Interactions were tested for association of risk factors to atherosclerosis among patients and controls.

a Seven vascular areas were assessed for atherosclerosis by electrocardiogram, ankle-arm index, right and left carotid and femoral intima-media thickness and abdominal aorta plaques.

b CVE included stroke, coronary artery disease or peripheral artery disease.

c Pack-years of smoking were calculated as number of cigarette packs (20 cig/pack) per day multiplied by number of years smoking.

d Increased alcohol intake was defined as >12 units/week.

e Physical inactivity was defined as activity less than 60 min/week.

f Increased WHR was defined as ⩾0.85 in females and ⩾0.90 in males.

The association of several risk factors with staging of atherosclerosis differed between patients and controls when interactions were applied (Table 3).

## Discussion

To our knowledge, our study of young and middle-aged ischaemic stroke patients and controls is the first one to identify predictors of numeric staging of atherosclerosis by detailed ultrasound diagnostics of carotid- and femoral arteries, the abdominal aorta and leg arteries, and by evaluation of ischaemic ECG signs. Previous studies have only assessed the impact of vascular risk factors restricted to one or two vascular areas, most commonly to carotid IMT, or to carotid and femoral IMT.^7,8,10,25^

We found that obesity, smoking, dyslipidaemia, and hypertension were the most frequent modifiable RFs among young stroke patients without sex differences, as also found in several European studies of young stroke.^6,32–34^ Notably, the risk factor burden in this study, and prevalence of atherosclerosis in our previous study was found equally high among young and middle-aged male patients and controls, and among middle-aged female patients and controls.^2^ A retrospective case-control study of patients ⩽40 years with premature CAD, and gender-matched controls reported dyslipidaemia, smoking, hypertension and obesity as more significantly associated in patients as compared to controls.^35^

In our final combined regression analysis, strongest risk factors related to numeric staging of atherosclerosis were age, hypertension, diabetes mellitus, smoking and increased BMI. The Bogalusa Heart Study showed that hypertension, dyslipidaemia, smoking, BMI, and an increased number of RFs were related to the extent of atherosclerosis in the aorta and coronary arteries in even younger individuals aged 2-39 years, who died due to trauma.^36^ Obesity, often defined as BMI ⩾30 kg/m^2^ in literature, associated with young stroke was found weak or absent when adjusted for vascular RFs in other studies.^37,38^ By contrast, one study found that increased BMI in childhood and adolescence was associated with young stroke.^39^ As BMI reflects body size rather than fat distribution,^40^ WHR seems more strongly associated with risk of stroke, rather than BMI.^41^ We found also atherosclerotic staging associated with increased WHR, but after adjustment for all variables, the finding became non-significant. Among stroke patients, WHR showed a positive trend. As majority of missing WHR had increased BMI, we might have underestimated its association with atherosclerosis in our study. In a meta-analysis of 58 prospective studies, 1 SD increase in BMI and WHR was associated with higher risk for CAD and ischaemic stroke for study participants aged 40-59 years, whereas the hazard ratio (HR) attenuated for older subjects (⩾70 years).^42^ The association between WHR and ischaemic stroke was stronger than that of BMI and ischaemic stroke (HR 1.25 vs 1.20) adjusted for age, sex and smoking.^42^

EAT and VAT were associated with increased numeric staging of atherosclerosis among our study-participants, but turned non-significant after adjustment for all variables. By contrast, other studies have found EAT and VAT to be predictors of CVD.^43–45^ SAT was not associated with atherosclerosis in any performed analysis, and we therefore did not evaluate SAT as risk factor for atherosclerosis. The Framingham Heart Study reported stronger associations of VAT with metabolic risk factors than with SAT.^46^

Physical inactivity was also significantly associated with increased numeric staging of atherosclerosis in the regression analysis done separately for patients and controls. Physical inactivity has been associated with obesity and worse cardiovascular profile, increasing the risk of ischaemic stroke (OR 5.9),^37^ and the fact that obesity provides an increased risk for earlier development of hypertension and diabetes mellitus.^47^

Finally, we found high risk factor matching regarding physical inactivity and smoking between patients and their partners as controls, indicating that couples share some habits as noted in genetic studies.^48^

Our study confirmed high prevalence of vascular risk factors and atherosclerosis among young and middle-aged stroke patients and controls, indicating necessity of both primary and secondary intervention in a population where CVEs are most common causes of death and hospital admissions.^19–21^ The primary goal may be achieved by educating individuals for a healthy lifestyle, and initiation of early treatment of asymptomatic individuals who are at high risk. As young stroke patients are at high risk for new CVE and earlier mortality, mainly due to atherosclerosis,^2,23^ they should be aimed at for aggressive secondary treatment of modifiable RFs.

### Strengths and limitations

Strengths of the present study are the population-based design, standardized diagnostic work-up of multiple RFs, and a comprehensive ultrasound protocol permitting numeric staging of atherosclerosis. Limitations are due to the necessity of age and sex matched analysis, which reduced the number of participants in each group, and thereby probably reduced the ability for reporting “statistically significant” results. But our findings seem to be in line with previous studies for stroke patients and young trauma victims, showing high prevalence of atherosclerosis related to vascular risk factors.^6,32,34,36^

We assessed only the prevalence of RFs, not the duration or severity that would be more actual for long-term data to show RFs change after treatment intervention, and its effects on atherosclerosis. Uncertainty about history of dyslipidaemia and diabetes mellitus was substantiated by blood tests among patients but not among controls, which resulted in an expected underestimation of its prevalence among controls. Dyslipidemia was not found significant in the final Poisson regression analysis, but was very frequent among patients, and we regard dyslipidemia as an important risk factor.

## Conclusion

This study expanded the knowledge about present atherosclerosis among young and middle-aged ischaemic stroke patients and controls by targeted individual artery wall diagnostics. We found a high burden of vascular RFs in both groups. No significant differences in the burden of risk factors among young and middle-aged males and middle-aged females emphasize the need for early primary prevention including health education, and early treatment of modifiable risk factors to avoid further progression of atherosclerosis. Main modifiable risk factors were hypertension, diabetes mellitus, pack-years of smoking, and a high BMI. We regard also dyslipidaemia as a well-known vascular risk factor, although we could not find this in our study.

## Supplemental Material

sj-docx-1-eso-10.1177_23969873221098582 – Supplemental material for Vascular risk factors and staging of atherosclerosis in patients and controls: The Norwegian Stroke in the Young Study Click here for additional data file. Supplemental material, sj-docx-1-eso-10.1177_23969873221098582 for Vascular risk factors and staging of atherosclerosis in patients and controls: The Norwegian Stroke in the Young Study by Beenish Nawaz, Annette Fromm, Halvor Øygarden, Geir Egil Eide, Sahrai Saeed, Rudy Meijer, Michiel L Bots, Kristin Modalsli Sand, Lars Thomassen, Halvor Næss and Ulrike Waje-Andreassen in European Stroke Journal

sj-docx-2-eso-10.1177_23969873221098582 – Supplemental material for Vascular risk factors and staging of atherosclerosis in patients and controls: The Norwegian Stroke in the Young Study Click here for additional data file. Supplemental material, sj-docx-2-eso-10.1177_23969873221098582 for Vascular risk factors and staging of atherosclerosis in patients and controls: The Norwegian Stroke in the Young Study by Beenish Nawaz, Annette Fromm, Halvor Øygarden, Geir Egil Eide, Sahrai Saeed, Rudy Meijer, Michiel L Bots, Kristin Modalsli Sand, Lars Thomassen, Halvor Næss and Ulrike Waje-Andreassen in European Stroke Journal

sj-docx-3-eso-10.1177_23969873221098582 – Supplemental material for Vascular risk factors and staging of atherosclerosis in patients and controls: The Norwegian Stroke in the Young Study Click here for additional data file. Supplemental material, sj-docx-3-eso-10.1177_23969873221098582 for Vascular risk factors and staging of atherosclerosis in patients and controls: The Norwegian Stroke in the Young Study by Beenish Nawaz, Annette Fromm, Halvor Øygarden, Geir Egil Eide, Sahrai Saeed, Rudy Meijer, Michiel L Bots, Kristin Modalsli Sand, Lars Thomassen, Halvor Næss and Ulrike Waje-Andreassen in European Stroke Journal
