# Supplementary material for: Vascular risk factors and staging of atherosclerosis in patients and controls: The Norwegian Stroke in the Young Study
Source: Eur Stroke J. 2022 May 10;7(3):289–98. doi: 10.1177/23969873221098582 (PMC9446327; doi:10.1177/23969873221098582)
Supplement: sj-docx-1-eso-10.1177_23969873221098582 – Supplemental material for Vascular risk factors and staging of atherosclerosis in patients and controls: The Norwegian Stroke in the Young Study [file sj-docx-1-eso-10.1177_23969873221098582.docx]

**Supplementary Figure 1:** Flow chart about inclusion- and exclusion criteria, and methods applied for patients and controls included into the Norwegian Stroke in the Young Study from 2010 – 2015

**Patients**

**n = 385**

**Controls**

**n = 260**

**Blood tests**

Cholesterol, triglycerides, glucose and other blood routine samples for patients only

**Exclusion criteria**

Serious co-morbidity or limited co-operation

**Standardized questionnaire**

- History of cardiovascular events, hypertension, dyslipidaemia, diabetes mellitus
- Use of medication for hypertension, dyslipidaemia, diabetes mellitus
- Tobacco use and alcohol intake
- Physical activity

**Diagnostics**

- Blood pressure measurements
- Body mass index and waist-hip ratio measurements
- Electrocardiography
- Ankle-arm index
- Standardized ultrasound measurements of
- Carotid and femoral intima-media thickness
- Abdominal aorta plaques
- Adipose tissue: epicardial, visceral and subcutaneous fat measurements
- Ankle-arm index

**Inclusion criteria**

Patients’ partners and/or ex-partner
at age ≥ 18 years

Included after patients’ and then own consent

**Inclusion criteria**

Acute ischaemic stroke
at age 15-60 years

Included after consent

**Exclusion criteria**

Stroke caused by trauma, sinus venous thrombosis, sepsis or procedure
