# Supplementary material for: Vascular risk factors and staging of atherosclerosis in patients and controls: The Norwegian Stroke in the Young Study
Source: Eur Stroke J. 2022 May 10;7(3):289–98. doi: 10.1177/23969873221098582 (PMC9446327; doi:10.1177/23969873221098582)
Supplement: sj-docx-2-eso-10.1177_23969873221098582 – Supplemental material for Vascular risk factors and staging of atherosclerosis in patients and controls: The Norwegian Stroke in the Young Study [file sj-docx-2-eso-10.1177_23969873221098582.docx]

**Supplementary Table 1** Prevalence of risk factors among young and middle-aged stroke patients in the Norwegian Stroke in the Young Study

|  |  | **Young aged ≤49 years** | | | |  | **Middle-aged ≥50 years** | | | |  |
| --- | --- | --- | --- | --- | --- | --- | --- | --- | --- | --- | --- |
| **Risk factors** | **NA, *n*** | **All, *n* (%) 152 (39.5)** | **Male, n (%) 94 (61.8)** | **Female, n (%) 58 (38.2)** | **P-Value Sex *** | **NA, *n*** | **All n (%) 233 (60.5)** | **Male, n (%) 170 (73.0)** | **Female, n (%) 63 (27.0)** | **P-value Sex *** | **P-value* Age group** |
| Prior CVE^a)^ | 0 | 13 (8.6) | 8 (61.5) | 5 (38.5) | 0.981 | 0 | 36 (15.4) | 27 (75.0) | 9 (25.0) | 0.765 | **0.047** |
| Hypertension^b)^ | 0 | 65 (42.8) | 46 (70.8) | 19 (29.2) | **0.050** | 0 | 173 (74.2) | 125 (72.3) | 48 (27.7) | 0.680 | **<0.001** |
| Diabetes mellitus^c)^ | 0 | 11 (7.2) | 6 (54.5) | 5 (45.5) | 0.605 | 0 | 33 (14.2) | 28 (84.8) | 5 (15.2) | 0.097 | **0.037** |
| Dyslipidaemia^d)^ | 0 | 96 (63.2) | 61 (63.5) | 35 (36.5) | 0.572 | 0 | 197 (84.5) | 145 (73.6) | 52 (82.5) | 0.605 | **<0.001** |
| Smoking^e)^ | 0 | 92 (60.5) | 59 (64.1) | 33 (35.9) | 0.472 | 0 | 176 (75.5) | 130 (76.5) | 46 (26.4) | 0.586 | **0.002** |
| Alcohol^f)^ | 2 | 13 (8.7) | 12 (92.3) | 1 (7.7) | **0.018** | 2 | 25 (10.8) | 22 (88.0) | 3 (12.0) | 0.069 | 0.493 |
| Physical inactivity^g)^ | 0 | 28 (18.4) | 21 (75.0) | 7 (25.0) | 0.113 | 0 | 43 (18.5) | 33 (76.7) | 10 (23.3) | 0.536 | 0.993 |
| BMI ≥25 kg/m^2^ | 2 | 97 (64.7) | 66 (68.0) | 31 (32.0) | 0.066 | 1 | 157 (67.7) | 123 (78.3) | 34 (21.7) | **0.006** | 0.543 |
| Increased WHR^h)^ | 11 | 83 (58.9) | 56 (64.9) | 27 (32.5) | 0.092 | 15 | 171 (78.4) | 131 (76.6) | 40 (23.4) | **0.009** | **<0.001** |
| Increased EAT^i)^ | 2 | 77 (51.3) | 50 (54.3) | 27 (35.1) | 0.352 | 11 | 152 (68.5) | 112 (68.7) | 40 (67.8) | 0.897 | **0.001** |
| Increased VAT^j)^ | 4 | 64 (43.2) | 42 (65.6) | 22 (34.4) | 0.448 | 5 | 130 (57.0) | 95 (73.1) | 35 (26.9) | 0.947 | **0.009** |
| Increased SAT^j)^ | 5 | 50 (34.0) | 29 (58.0) | 21 (42.0) | 0.484 | 4 | 77 (33.6) | 51 (66.2) | 26 (33.8) | 0.105 | 0.938 |
| Total RFB^k)^ | 15 | 131 (95.6) | 84 (61.3) | 53 (38.7) | 0.544 | 24 | 209 (100.0) | 152 (72.7) | 57 (27.3) | 0.486 | **<0.001** |

*Abbreviations:* NA = not available*; n* = number of patients, CVE = cardiovascular events; BMI = body mass index; WHR = waist-hip ratio; EAT = epicardial adipose tissue; VAT = visceral abdominal adipose tissue; SAT = subcutaneous abdominal adipose tissue; RFB = risk factor burden

a) Prior CVE included stroke, coronary artery disease or peripheral artery disease; b) Hypertension was defined as known or diagnosed if blood pressure >140/90 mmHg; c) Diabetes mellitus was defined as known among patients and controls, or diagnosed by HbA1c >6.5% among patients only; d) Dyslipidaemia was defined as known among patients and controls, or diagnosed by blood tests among patients only; e). Smoking included ex-smokers and active smokers; f) Alcohol consumption is defined as ≥12 units/week; g) Physical inactivity was defined as activity less than 60 minutes/week; h) Increased WHR was defined as ≥0.85 in females and ≥0.9 in men; i). Increased EAT is defined as >0.5 cm; j). Increased VAT and SAT are based on 90^th^ percentile of sex-specific cut points from normal weight referent sample; k) number of risk factors present ≥1.

*Chi-square test.
