# Supplementary material for: Vascular risk factors and staging of atherosclerosis in patients and controls: The Norwegian Stroke in the Young Study
Source: Eur Stroke J. 2022 May 10;7(3):289–98. doi: 10.1177/23969873221098582 (PMC9446327; doi:10.1177/23969873221098582)
Supplement: sj-docx-3-eso-10.1177_23969873221098582 – Supplemental material for Vascular risk factors and staging of atherosclerosis in patients and controls: The Norwegian Stroke in the Young Study [file sj-docx-3-eso-10.1177_23969873221098582.docx]

**Supplementary Table 2** Similar risk factors in 257 couples^*^ in the Norwegian Stroke in the Young Study

|  | **Risk factors in 385 patients, *n* (%)** | **Risk factors in 260 controls, *n* (%)** | **NA,**  ***n*** | **Prevalent risk factors in 257 couples, *n* (%)** |
| --- | --- | --- | --- | --- |
| Prior CVE^a)^ | 49 (12.7) | 18 (6.9) | 0 | 5 (1.9) |
| Hypertension^b)^ | 238 (61.8) | 89 (34.4) | 1 | 63 (24.6) |
| Diabetes mellitus^c)^ | 44 (11.4) | 15 (5.8) | 0 | 3 (1.2) |
| Dyslipidaemia^d)^ | 293 (76.1) | 34 (13.1) | 0 | 26 (10.1) |
| Smoking^e)^ | 268 (69.6) | 169 (65.3) | 1 | 141 (55.1) |
| Alcohol^f)^ | 38 (10.0) | 8 (3.1) | 4 | 3 (1.2) |
| Physical inactivity^g)^ | 71 (18.4) | 28 (10.8) | 0 | 191 (74.3) |
| BMI ≥25 kg/m^2^ | 254 (66.5) | 152 (59.4) | 6 | 107 (42.6) |
| Increased WHR^h)^ | 254 (70.8) | 130 (51.0) | 25 | 95 (40.9) |
| Increased EAT^i)^ | 229 (61.6) | 147 (56.8) | 6 | 106 (42.2) |
| Increased VAT^j)^ | 194 (51.6) | 77 (29.7) | 4 | 44 (17.4) |
| Increased SAT^j)^ | 127 (33.8) | 72 (27.8) | 5 | 27 (10.7) |

*Abbreviations:* NA = missing couples; CVE = cardiovascular event; BMI = body-mass index; WHR = waist-hip ratio; EAT = epicardial adipose tissue; VAT = visceral abdominal adipose tissue; SAT = subcutaneous abdominal adipose tissue.

*There were 257 couples in total out of 385 patients and 260 controls, where 16 patients had two partners included (ex-partner/ partner) and three patients were excluded retrospectively but their partners remained in the study.

a) Prior CVE included stroke, coronary artery disease or peripheral artery disease; b) Hypertension was defined as known or diagnosed if blood pressure >140/90 mmHg; c) Diabetes mellitus was defined as known among patients and controls, or diagnosed by HbA1c >6.5% among patients only; d) Dyslipidaemia was defined as known among patients and controls, or diagnosed by blood tests among patients only; e) Smoking included ex-smokers and active smokers; f) Alcohol consumption is defined as ≥12 units/week; g) Physical inactivity was defined as activity less than 60 minutes/week; h) Increased WHR was defined as ≥0.85 in females and ≥0.9 in men; i). Increased EAT is defined as >0.5 cm; j). Increased VAT and SAT are based on 90^th^ percentile of sex-specific cut points from normal weight referent sample.
